# An Open Resource for Non-human Primate Imaging

**DOI:** 10.1016/j.neuron.2018.08.039

**Published:** 2018-10-10

**Authors:** Michael P. Milham, Lei Ai, Bonhwang Koo, Ting Xu, Céline Amiez, Fabien Balezeau, Mark G. Baxter, Erwin L.A. Blezer, Thomas Brochier, Aihua Chen, Paula L. Croxson, Christienne G. Damatac, Stanislas Dehaene, Stefan Everling, Damian A. Fair, Lazar Fleysher, Winrich Freiwald, Sean Froudist-Walsh, Timothy D. Griffiths, Carole Guedj, Fadila Hadj-Bouziane, Suliann Ben Hamed, Noam Harel, Bassem Hiba, Bechir Jarraya, Benjamin Jung, Sabine Kastner, P. Christiaan Klink, Sze Chai Kwok, Kevin N. Laland, David A. Leopold, Patrik Lindenfors, Rogier B. Mars, Ravi S. Menon, Adam Messinger, Martine Meunier, Kelvin Mok, John H. Morrison, Jennifer Nacef, Jamie Nagy, Michael Ortiz Rios, Christopher I. Petkov, Mark Pinsk, Colline Poirier, Emmanuel Procyk, Reza Rajimehr, Simon M. Reader, Pieter R. Roelfsema, David A. Rudko, Matthew F.S. Rushworth, Brian E. Russ, Jerome Sallet, Michael Christoph Schmid, Caspar M. Schwiedrzik, Jakob Seidlitz, Julien Sein, Amir Shmuel, Elinor L. Sullivan, Leslie Ungerleider, Alexander Thiele, Orlin S. Todorov, Doris Tsao, Zheng Wang, Charles R.E. Wilson, Essa Yacoub, Frank Q. Ye, Wilbert Zarco, Yong-di Zhou, Daniel S. Margulies, Charles E. Schroeder

**Affiliations:** 1 Center for the Developing Brain, Child Mind Institute, New York, NY 10022, USA; 2 Center for Biomedical Imaging and Neuromodulation, Nathan S. Kline Institute for Psychiatric Research, Orangeburg, NY 10962, USA; 3 University of Lyon, Université Claude Bernard Lyon 1, INSERM, Stem Cell and Brain Research Institute U1208, Lyon, France; 4 Institute of Neuroscience, Newcastle University, Newcastle upon Tyne NE1 7RU, UK; 5 Department of Neuroscience, Icahn School of Medicine at Mount Sinai, New York, NY 10029, USA; 6 Institut des Sciences Cognitives – Marc Jeannerod, UMR5229, CNRS-Université de Lyon, Lyon, France; 7 Institut de Neurosciences de la Timone, CNRS & Aix-Marseille Université, UMR 7289, Marseille, France; 8 Key Laboratory of Brain Functional Genomics (Ministry of Education & Science and Technology Commission of Shanghai Municipality), School of Life Sciences, East China Normal University, Shanghai 200062, China; 9 Donders Institute for Brain, Cognition and Behavior, Radboud University Nijmegen, 6525 EN Nijmegen, Netherlands; 10 NeuroSpin, CEA, INSERM U992, Université Paris-Saclay, 91191 Gif-sur-Yvette, France; 11 Centre for Functional and Metabolic Mapping, The University of Western Ontario, London, ON N6A 3K7, Canada; 12 Department of Behavior Neuroscience, Department of Psychiatry, Advanced Imaging Research Center, Oregon Health and Science University, Portland, OR, USA; 13 Department of Radiology, Icahn School of Medicine at Mount Sinai, New York, NY 10029, USA; 14 Laboratory of Neural Systems, The Rockefeller University, New York, NY, USA; 15 Center for Neural Science, New York University, New York, NY 10023, USA; 16 INSERM, U1028, CNRS UMR5292, Lyon Neuroscience Research Center, Lyon, France; 17 Center for Magnetic Resonance Research, University of Minnesota Medical School, Minneapolis, MN 55455, USA; 18 Laboratory of Brain and Cognition, National Institute of Mental Health, Bethesda, MD 20892, USA; 19 Princeton Neuroscience Institute, Princeton University, Princeton, NJ 08540, USA; 20 Netherlands Institute for Neuroscience, Royal Netherlands Academy of Arts and Sciences, 1105 BA Amsterdam, the Netherlands; 21 Department of Psychiatry, Amsterdam UMC, University of Amsterdam, 1105 AZ Amsterdam, the Netherlands; 22 Shanghai Key Laboratory of Brain Functional Genomics, School of Psychology and Cognitive Science, Key Laboratory of Brain Functional Genomics (Ministry of Education), East China Normal University, Shanghai 200062, China; 23 Shanghai Key Laboratory of Magnetic Resonance, East China Normal University, Shanghai 200062, China; 24 NYU-ECNU Institute of Brain and Cognitive Science at NYU Shanghai, Shanghai 200062, China; 25 Section on Cognitive Neurophysiology and Imaging, National Institute of Mental Health, Bethesda, MD 20892, USA; 26 Neurophysiology Imaging Facility, National Institute of Mental Health, National Institute of Neurological Disorders and Stroke, National Eye Institute, Bethesda, MD 20892, USA; 27 Wellcome Centre for Integrative Neuroimaging, Centre for Functional MRI of the Brain (FMRIB), Nuffield Department of Clinical Neurosciences, John Radcliffe Hospital, University of Oxford, Oxford OX3 9DU, UK; 28 McConnell Brain Imaging Centre, Montreal Neurological Institute, Departments of Neurology, Neurosurgery, and Biomedical Engineering, McGill University, Montreal, QC H3A 0G4, Canada; 29 California National Primate Research Center, Davis, CA 95616, USA; 30 Department of Neurology, School of Medicine, University of California, Davis, CA 95616, USA; 31 McGovern Institute for Brain Research, Massachusetts Institute of Technology, Cambridge, MA 02139, USA; 32 Department of Biology and Helmholtz Institute, Utrecht University, 35 84 CH Utrecht, The Netherlands; 33 Department of Biology, McGill University, Montreal, QC H3A 1BA, Canada; 34 Department of Integrative Neurophysiology, Center for Neurogenomics and Cognitive Research, Vrije Universiteit, 1081 HV Amsterdam, the Netherlands; 35 Wellcome Centre for Integrative Neuroimaging, Department of Experimental Psychology, University of Oxford, Oxford OX1 3AQ, UK; 36 Developmental Neurogenomics Unit, National Institute of Mental Health, Bethesda, MD 20892, USA; 37 Brain Mapping Unit, Department of Psychiatry, University of Cambridge, Cambridge CB2 0SZ, UK; 38 Divisions of Neuroscience and Cardiometabolic Health, Oregon National Primate Research Center, Beaverton, OR, USA; 39 Department of Human Physiology, University of Oregon, Eugene, OR, USA; 40 Department of Computation and Neural Systems, California Institute of Technology, Pasadena, CA 91125, USA; 41 Institute of Neuroscience, Key Laboratory of Primate Neurobiology, Shanghai Institutes for Biological Sciences, Chinese Academy of Sciences, Shanghai, China; 42 Krieger Mind/Brain Institute, Department of Neurosurgery, Johns Hopkins University, Baltimore, MD 21287, USA; 43 Max Planck Research Group for Neuroanatomy and Connectivity, Max Planck Institute for Human Cognitive and Brain Sciences, 04103 Leipzig, Germany; 44 Centre national de la recherche scientifique, CNRS UMR 7225, Institut du Cerveau et de la Moelle épinière, 75013 Paris, France; 45 Department of Neurological Surgery, Columbia University College of Physicians and Surgeons, New York, NY 10032, USA; 46 Department of Psychiatry, Columbia University College of Physicians and Surgeons, New York, NY 10032, USA; 47 Biomedical MR Imaging and Spectroscopy Group, Center for Image Sciences, University Medical Center Utrecht, Utrecht, The Netherlands; 48 Institute for Future Studies, Stockholm, Sweden; 49 Centre for Cultural Evolution & Department of Zoology, Stockholm University, Stockholm, Sweden; 50 Centre for Social Learning and Cognitive Evolution, School of Biology, University of St. Andrews, St. Andrews, UK

## Abstract

Non-human primate neuroimaging is a rapidly growing area of research that promises to transform and scale translational and cross-species comparative neuroscience. Unfortunately, the technological and methodological advances of the past two decades have outpaced the accrual of data, which is particularly challenging given the relatively few centers that have the necessary facilities and capabilities. The PRIMatE Data Exchange (PRIME-DE) addresses this challenge by aggregating independently acquired non-human primate magnetic resonance imaging (MRI) datasets and openly sharing them via the International Neuroimaging Data-sharing Initiative (INDI). Here, we present the rationale, design, and procedures for the PRIME-DE consortium, as well as the initial release, consisting of 25 independent data collections aggregated across 22 sites (total = 217 non-human primates). We also outline the unique pitfalls and challenges that should be considered in the analysis of non-human primate MRI datasets, including providing automated quality assessment of the contributed datasets.

## Highlights

• Openly shared, large non-human primate neuroimaging data resource
• Multiple imaging modalities contributed from investigators around the world
• Quality assessments of the dataset
• Discussed pitfalls and challenges in analyzing the non-human primate MRI data

## Teaser

The PRIMatE Data Exchange (PRIME-DE) consortium is an open science resource for the neuroimaging community aiming to facilitate efforts to map the non-human primate connectome. It aggregates and shares anatomical, functional, and diffusion MRI datasets from laboratories throughout the world.

## Introduction

Translational, comparative neuroscience research enables a bridging of knowledge gaps across species as well as invasive and noninvasive approaches. A growing body of research has documented the utility of magnetic resonance imaging (MRI) technologies to support *in vivo* examination of brain organization and function in non-human primates (Vanduffel et al., 2014, Rilling, 2014, Van Essen and Glasser, 2014, Zhang et al., 2013, Shmuel and Leopold, 2008, Schwiedrzik et al., 2015). Recent work has demonstrated the ability to recapitulate findings from gold-standard invasive methodologies (Ghahremani et al., 2017, Donahue et al., 2016, Grayson et al., 2016). This work also provides novel insights into the organizational principles of the non-human primate (NHP) connectome (Goulas et al., 2017, Hutchison and Everling, 2014, Hutchison et al., 2011, Vincent et al., 2007) and cross-species comparative connectomics (Hutchison et al., 2012, Hutchison et al., 2015, Miranda-Dominguez et al., 2014, Mars et al., 2011, Seidlitz et al., 2018a), which are possible only through *in vivo* studies. These advances are timely given the growing prominence of large-scale national and international initiatives focused on advancing our understanding of human brain organization and the ability to generate novel therapeutics for neurology and psychiatry (Bargmann and Newsome, 2014).

Despite the clear demonstrations of feasibility and utility, the field of non-human primate neuroimaging is still developing. Numerous unique challenges related to the acquisition and processing of non-human primate data are still being addressed (e.g., Seidlitz et al., 2018b, Hutchison and Everling, 2012), and the potential for broad reaching cross-species studies remains unexploited. Perhaps most challenging is the limited availability of data.

Here, we introduce the PRIMatE Data Exchange (PRIME-DE) to create an open science resource for the neuroimaging community that will facilitate the mapping of the non-human primate connectome. To accomplish this, we aggregate a combination of anatomical, functional, and diffusion MRI datasets from laboratories throughout the world and make these data available to the scientific community. It merits emphasis that PRIME-DE supports an ongoing process that will remain open to new contributions of data from macaques and other non-human primate species.

## Results

### Overview

At present, PRIME-DE contains 25 collections aggregated across 22 sites; to date, data from 217 primates are included (see Table 1 for information on each institution). Contributions will continue to be accepted and shared on a rolling basis.

**Table 1:** Experimental Design

	Investigators	Species^a^	Subjects	State	Contrast Agent	Structural T1	Structural T2	Resting State fMRI	Naturalistic Viewing fMRI	Task fMRI	Field map	Diffusion MRI
AMU	Belin, Brochier, Sein	MM	4	Anesthetized	No	✔	✔	–	–	–	–	✔
Caltech	Rajimehr, Tsao	MM	2	Awake	Yes	–	–	–	96 min	–	–	–
ECNU (C)	Aihua Chen	MM	10	Anesthetized	No	✔	–	–	–	–	–	–
ECNU (K)^b^	Kwok, Zhou	MM	4	Anesthetized	No	✔	✔	8 min	–	–	–	✔
Institute of Neuroscience (IoN)	Wang	MM, MF	8	Anesthetized	No	✔	–	20–40 min	–	–	✔	–
Institut des Sciences Cognitives Marc Jeannerod	Ben Hamed, Hiba	MM	8	Anesthetized/Awake	Yes	✔	–	✔	–	✔	–	✔
Lyon Neuroscience Research Center	Hadj-Bouziane, Meunier, Guedj	MM	1	Anesthetized/Awake	Yes/No	✔	✔	13 min	–	–	–	–
McGill University	Mok, Rudko, Shmuel	MM, MF	3	Anesthetized	No	✔	✔	–	–	–	–	–
Mount Sinai (P)	Croxson, Fleysher	MM, MF	9	Anesthetized	No	✔	✔	43 min	–	–	✔	✔
Mount Sinai (S)	Croxson, Fleysher, Froudist-Walsh, Damatac, Nagy	MM	5	Anesthetized	No	✔	✔	–	–	–	–	✔
NKI	Schroeder, Milham	MM	2	Anesthetized/Awake	Yes/No	✔		76–155 min	55–345 min	–	–	–
NIMH (L)^c^	Leopold, Russ	MM	3	Awake	Yes	✔	✔	30–150 min	170 min	–	–	–
NIMH (M)^c^^,^^d^	Messinger, Jung, Seidlitz, Ungerleider	MM	3	Anesthetized/Awake	Yes	✔	–	10−15 min	–	–	–	–
Netherlands Institute for Neuroscience (NIN)	Klink, Roelfsema	MM	2	Anesthetized	No	✔	✔	9.7 min	–	–	–	–
NeuroSpin	Jarraya, Dehaene	MM	3	Anesthetized	Yes/No	✔	–	✔	–	–	–	–
Newcastle	Petkov, Nacef, Thiele, Poirier, Balezeau, Griffiths, Schmid, Rios	MM	14	Anesthetized/Awake	No	✔	✔	21.6 min	–	–	–	–
OHSU	Sullivan, Fair	MM	2	Anesthetized	Yes/No	✔	✔	480 min	–	–	–	–
Princeton	Kastner, Pinsk	MM	2	Anesthetized		✔	✔	–	–	–	✔	✔
Rockefeller	Schwiedrzik, Freiwald, Zarco	MM, MF	6	Anesthetized	Yes	✔	–	80 min	–	–	✔	
SBRI	Procyk, Wilson, Amiez	MM, MF	22	Anesthetized	No	✔	✔	✔	–	–	–	–
UC Davis	Baxter, Croxson, Morrison	MM	19	Anesthetized	No	✔	✔	13.5 min	–	–	✔	✔
Univ. of Minnesota (UMN)	Yacoub, Harel	M	2	Anesthetized	–	✔	–	27 min	–	–	✔	✔
Univ. of Oxford^c^	Sallet, Mars, Rushworth	MM	20	Anesthetized	No	✔	–	53.43 min	–	–	–	–
NIN Primate Brain Bank/Utrecht University	Navarrete, Blezer, Todorov, Lindenfors, Laland, Reader	Multiple^a^	51	Post-mortem	Yes/No	✔	–	–	–	–	–	–
Univ. of Western Ontario (UWO)	Everling, Menon	MM	12	Anesthetized	No	✔	✔	60 min	–	–	–	–

General information about PRIME-DE data collections contributed prior to the time of publication. For usage agreement, CC-BY-NC-SA: Creative Commons – Attribution-NonCommercial Share Alike, Standard INDI data sharing policy, prohibits use of the data for commercial purposes; DUA: Data Usage Agreement, users must complete a DUA prior to gaining access to the data. For species information, MM: Macaca mulatta; MF: Macaca fascicularis; M: Macaca.

a Detailed species information is available on the PRIME-DE site and in Navarrete et al., 2018

b ECNU (K) provided magnetic resonance spectroscopy

c The usage agreement is DUA for those sites, CC-BY-NC-SA for all other sites

d NIMH (M) provided cortical thickness and brain template

To promote usage of a standardized data format, we organized all data using the Brain Imaging Data Structure (BIDS) format (Gorgolewski et al., 2017). All PRIME-DE datasets can be accessed through the PRIME-DE site (http://fcon_1000.projects.nitrc.org/indi/indiPRIME.html). Prior to downloading the data, users are required to establish a user account on NITRC and register with the International Neuroimaging Data-sharing Initiative (INDI; anticipated time: <1 min).

### MRI Data

With one exception, for each of the PRIME-DE collections, at least one structural MRI (sMRI) is available for each unique ID number (see Table 1). Eighteen of the collections contain at least one corresponding resting-state functional MRI (R-fMRI) dataset, and three of the collections contain naturalistic viewing fMRI (NV-fMRI). In addition, one collection from the National Institutes of Mental Health (NIMH (M)) also provided cortical thickness data and R-fMRI data aligned to an anatomical template. Corresponding diffusion MRI (dMRI) datasets are available for nine collections. Field map images for fMRI correction are available for six collections. Consistent with its popularity in the imaging community and prior usage in INDI efforts, the NIFTI file format was selected for storage of the PRIME-DE MRI datasets. Table 2 lists the specific MRI scanners and head coils utilized for each collection. Specific MRI sequence parameters for the various data collections are summarized in Tables S1, S2, S3, and S4 and detailed on the PRIME-DE website. Across collections, R-fMRI acquisition durations varied from 8 to 155 min per subject. In two collections, subjects were in an awake state. In five collections, subjects were scanned both awake and under anesthesia. One collection scanned 51 post-mortem specimens. In the remaining 17 collections, subjects were scanned under anesthesia. For the three collections with NV-FMRI, acquisition durations varied from 55 to 375 min. See Figures 3 and 4 for example structural and functional images from the different sites aligned in a common space.

**Table 2:** Scanner Information

Site	Manufacturer	Model	Field Strength (T)	Head coil # channels
AMU	Siemens	Prisma	3	Body transmit array, 11 cm loop receiving coil
Caltech	Siemens	Tim Trio	3	8
ECNU (C)	Siemens	Tim Trio	3	–
ECNU (K)	Siemens	Tim Trio	3	1-channel surface coil
Institute of Neuroscience (IoN)	Siemens	Tim Trio	3	8-channel phased-array transceiver coils
Institut des Sciences Cognitives Marc Jeannerod	Siemens	Sonata/Prisma	1.5/3	8-channel custom head coils/association of independent circular coils
Lyon Neuroscience Research Center	Siemens	Sonata/Prisma	1.5/3	Custom-made 10 cm loop receiving coil 2 × L11 and 1 × L7 Siemens loop-receiving coil
McGill University	Siemens	Tim Trio	3	Custom-made 8-channel phased-array receive coil
Mount Sinai (P)	Philips	Achieva	3	Single loop receive coil (T1 and T2) 4-channel phased-array receive, transmit through body coil (resting state and diffusion)
Mount Sinai (S)	Siemens	Skyra	3	8-channel phased-array receive with a single loop transmit
NKI	Siemens	Tim Trio	3	Custom-made 8-channel phased-array receive coil (KU Leuven) with a custom 16-channel pre-amplifier (MRcoils)
NIMH (L)	Bruker	BiospecVertical	4.7	8
NIMH (M)	Bruker	BiospecVertical	4.7	1–4
Netherlands Institute for Neuroscience (NIN)	Philips	Ingenia	3	Custom-made 8-channel phased-array receive coil (KU Leuven) with a custom 16-channel pre-amplifier (MRcoils).
NeuroSpin	Siemens	Tim Trio/PrismaFit	3	1chTxRxcoil/1Tx-8Rxchcoil
Newcastle	Bruker	Vertical Bruker	4.7	4–8
OHSU	Siemens	Tim Trio	3	Knee coil 15 channel
Princeton	Siemens	Prisma VE11C	3	Siemens Loop Coil, Large (11 cm)
Rockefeller	Siemens	TIM Trio + AC88 gradient	3	8-channel phased-array receive with a single-loop transmit
SBRI	Siemens	Sonata/Prisma	1.5/3	Custom made 10 cm loop receiving coil 2 × L11 and 1 × L7 Siemens loop receiving coil
UC Davis	Siemens	Skyra	3	4
Univ. of Minnesota (UMN)	Siemens	SyngoB17	7	16-channel transmit/receive + 6 receive only
Univ. of Oxford	–	–	3	A four-channel phased-array coil
NIN Primate Brain Bank/Utrecht University	Varian/Siemens	Small-bore scanner/Magnetom trio	9.4/3	–
Univ. of Western Ontario (UWO)	Siemens	Magnetom	7	Custom-made 24-channel phased-array receive coil with an 8-channel transmit coil

Information on scanner and head coil for PRIME-DE data collections contributed prior to the time of publication. Note that scanner information from University of Oxford is not reported due to an agreement made previously with the scanner manufacturer. For scan sequences, see also Tables S1, S2, S3, and S4.

### Data Licensing

Contributors to PRIME-DE will be able to set the sharing policy for their data in accord with their preferences and institutional requirements. For each sample, the contributor will set the sharing permissions for their data using one or more the following three policies:

(1) Creative Commons – Attribution-Non-Commercial Share Alike (CC-BY-NC-SA) (https://creativecommons.org/licenses/by-nc-sa/4.0/). Standard INDI data sharing policy. Prohibits use of the data for commercial purposes.
(2) Creative Commons – Attribution (CC-BY) (https://creativecommons.org/licenses/by/4.0/). Least restrictive data sharing policy.
(3) Custom Data Usage Agreement. Users must complete a data usage agreement (DUA) prior to gaining access to the data. Contributors can customize the agreement as they see fit, including determining whether or not signatures from authorized institutional official are required prior to executing the DUA. Note: this option was created to facilitate potential contributors whose institution requires completion of a formal interinstitutional agreement in order to share non-human primate data. Of note, one lesson learned from the human neuroimaging literature is that such agreements are not dissuasive, as is evidenced by the success of the Human Connectome Project (Van Essen et al., 2013) and the NKI-Rockland Sample (Nooner et al., 2012).

### Automated Quality Assessment

Consistent with the established policy of INDI, all data contributed to PRIME-DE was made available to users regardless of data quality; users should check data quality before inclusion in their analyses. The rationale of this decision has been the lack of consensus on optimal quality criteria in regards to specific measures or their combinations and cutoffs—a reality that is even more pronounced in non-human primate imaging given the variation in data quality and characteristics across scan protocols. Of note, a benefit of sharing data with differing levels of quality data is also important for those working to develop methods for evaluating, and at times overcoming, such variations.

**Figure 1:**
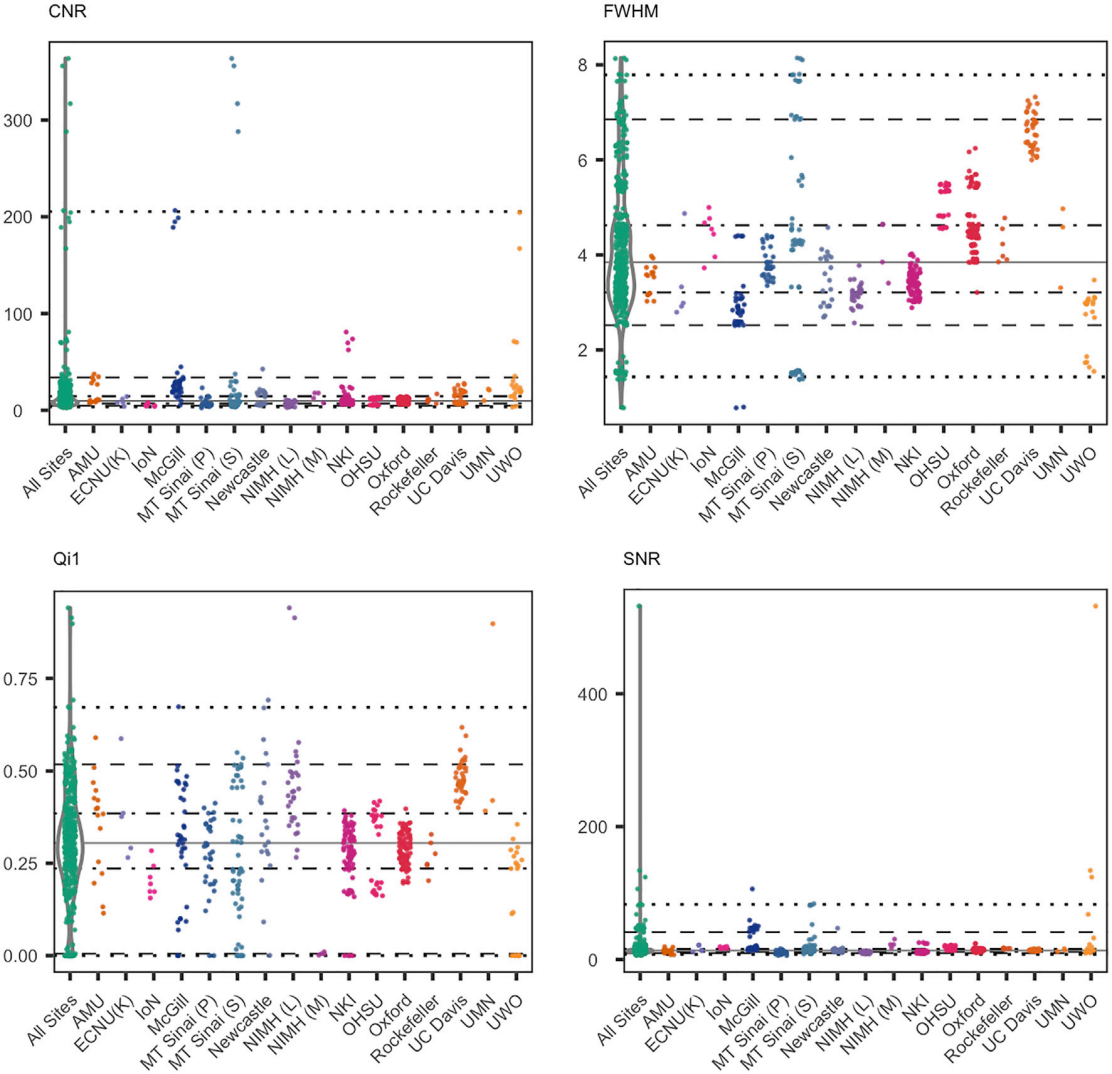
Spatial Quality Metrics for Morphometry MRI Datasets Spatial quality metrics include: contrast-to-noise ratio (CNR), smoothness of voxels indexed as full width at half maximum (FWHM), signal-to-noise ratio (SNR), and artifactual voxel detection (Qi1). See Table 3 for details on this and the other quality metrics released. The colored scatterplots illustrate the quality metrics distribution for each data collection. The violin plots on the left of each panel represent a kernel density estimation of the distribution across all data collections for each quality metric. Starting from the bottom: each horizontal line marks the 1^st^, 5^th^, 25^th^, 50^th^, 75^th^, 95^th^, and 99^th^ percentiles.

**Figure 2:**
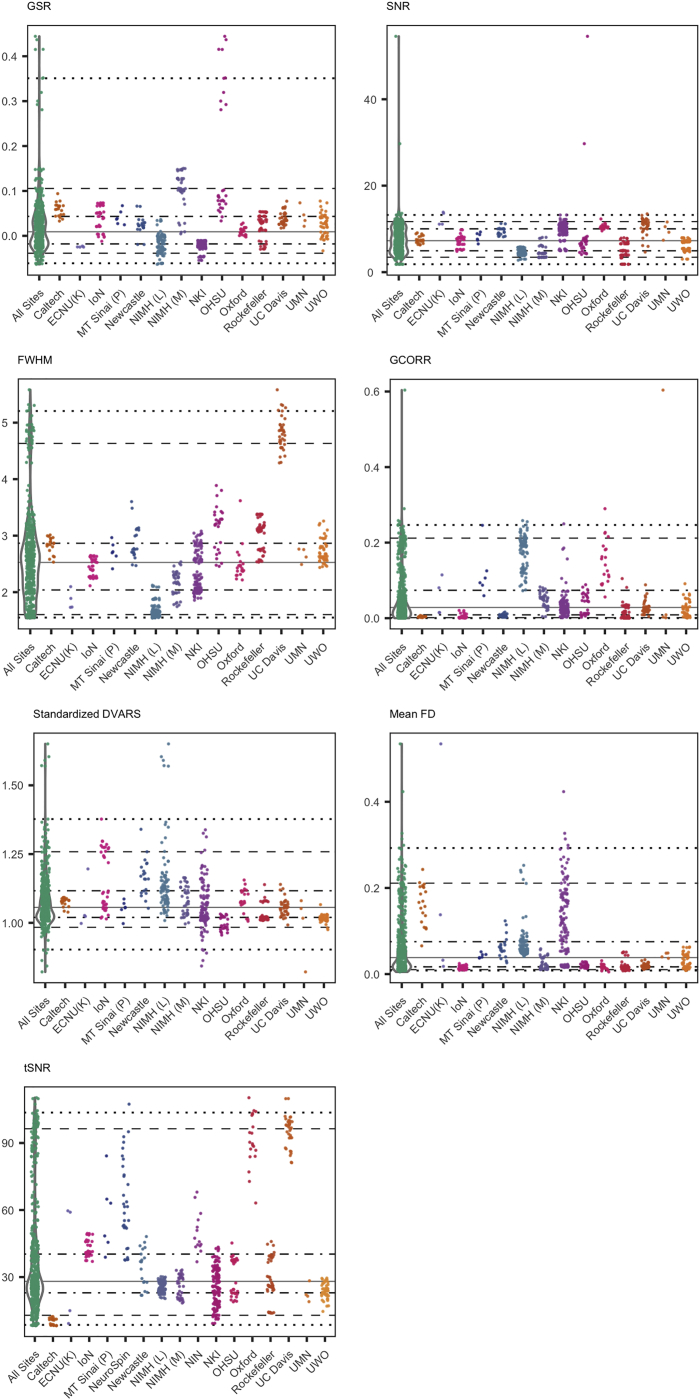
Spatial and Temporal Quality Metrics for Functional MRI Datasets Spatial quality metrics include: ghost-to-single ratio (GSR), smoothness of voxels indexed as full width at half maximum (FWHM), and signal-to-noise ratio (SNR). Temporal metrics are mean frame-wise displacement (Mean FD), standardized DVARS, global correlation (GCORR), and temporal signal-to-noise ratio (tSNR). See Table 3 for details on this and the other quality metrics released. The colored scatterplots illustrate the quality metrics distribution for each data collection. The violin plots on the left of each panel represent a kernel density estimation of the distribution across all data collections for each quality metric. Starting from the bottom: each horizontal line marks the 1^st^, 5^th^, 25^th^, 50^th^, 75^th^, 95^th^, and 99^th^ percentiles.

Following the tradition of recent INDI data-sharing consortia, a collection of automated, reference-free quality assurance measures, known as the Preprocessed Connectome Project Quality Assurance Protocol (PCP-QAP; Shehzad et al., 2015), is being made available with the PRIME-DE datasets. These measures focus on structural and temporal (when appropriate) aspects of the datasets. Table 3 provides a brief description of the measures included, and Figures 1 and 2 depict a subset of QAP results (Magnotta et al., 2006, Mortamet et al., 2009, Giannelli et al., 2010, Jenkinson et al., 2002, Friedman et al., 2006, Nichols, 2012). As would be expected, measures of head motion are notably smaller for sites using anesthetized scan sessions than for awake (NIMH (L), NIMH (M), NKI, Newcastle, Lyon Neuroscience Research Center). Importantly, the measures provided are not intended to be definitive for the field or all encompassing; rather, they are included to spur interest in the potential utility and further development of automated measures.

**Table 3:** Description of PCP QAP Measures

Spatial Metrics	Description	References
Contrast-to-noise ratio (CNR) (sMRI only)	M_GM_ intensity—M_WM_ intensity/SD_air_ intensity. Larger values reflect a better distinction between WM and GM.	Magnotta et al., 2006
Artifactual voxel detection (Qi1) (sMRI only)	Voxels with intensity corrupted by artifacts/voxels in the background. Larger values reflect more artifacts which likely due to motion or image instability.	Mortamet et al., 2009
Smoothness of Voxels (FWHM)^a^	Full width at half maximum of the spatial distribution of the image intensity values. Larger values reflect more spatial smoothing perhaps due to motion or technical differences.	Friedman et al., 2006
Signal-to-noise ratio (SNR)	M_GM_ intensity/SD_air_ intensity. Larger values reflect less noise.	Magnotta et al., 2006

**Temporal Metrics (fMRI and DTI only)**	**Description**	**References**

Ghost-to-Signal Ratio (GSR)^a^	M signal in the “ghost” image divided by the M signal within the brain. Larger values reflect more ghosting likely due to physiological noise, motion, or technical issues.	Giannelli et al., 2010
Mean frame-wise displacement- Jenkinson (meanFD)^b^	Sum absolute displacement changes in the x, y, and z directions and rotational changes around them. Rotational changes are given distance values based on changes across the surface of a 50 mm radius sphere. Larger values reflect more movement.	Jenkinson et al. 2002
Standardized DVARS^b^	Spatial SD of the data temporal derivative normalized by the temporal SD and autocorrelation. Larger values reflect larger frame-to-frame differences in signal intensity due to head motion or scanner instability.	Nichols, 2012
Global Correlation (GCORR)^b^	M correlation of all combinations of voxels in a time series. Illustrates differences between data due to motion/physiological noise. Larger values reflect a greater degree of spatial correlation between slices, which may be due to head motion or “signal leakage” in simultaneous multi-slice acquisitions.	–

Here, we provide a brief description of the Preprocessed Connectome Project Quality Assessment Protocol. These measures have been computed for all structural MRI (sMRI) and resting-state functional MRI (R-fMRI) datasets in PRIME-DE. The table was adopted from Di Martino et al. (2017).

a For R-fMRI data, these metrics are computed on mean functional data

b For R-fMRI, these metrics are computed on time series data. M, mean; GM, gray matter; WM, white matter; SD, standard deviation

**Figure 3:**
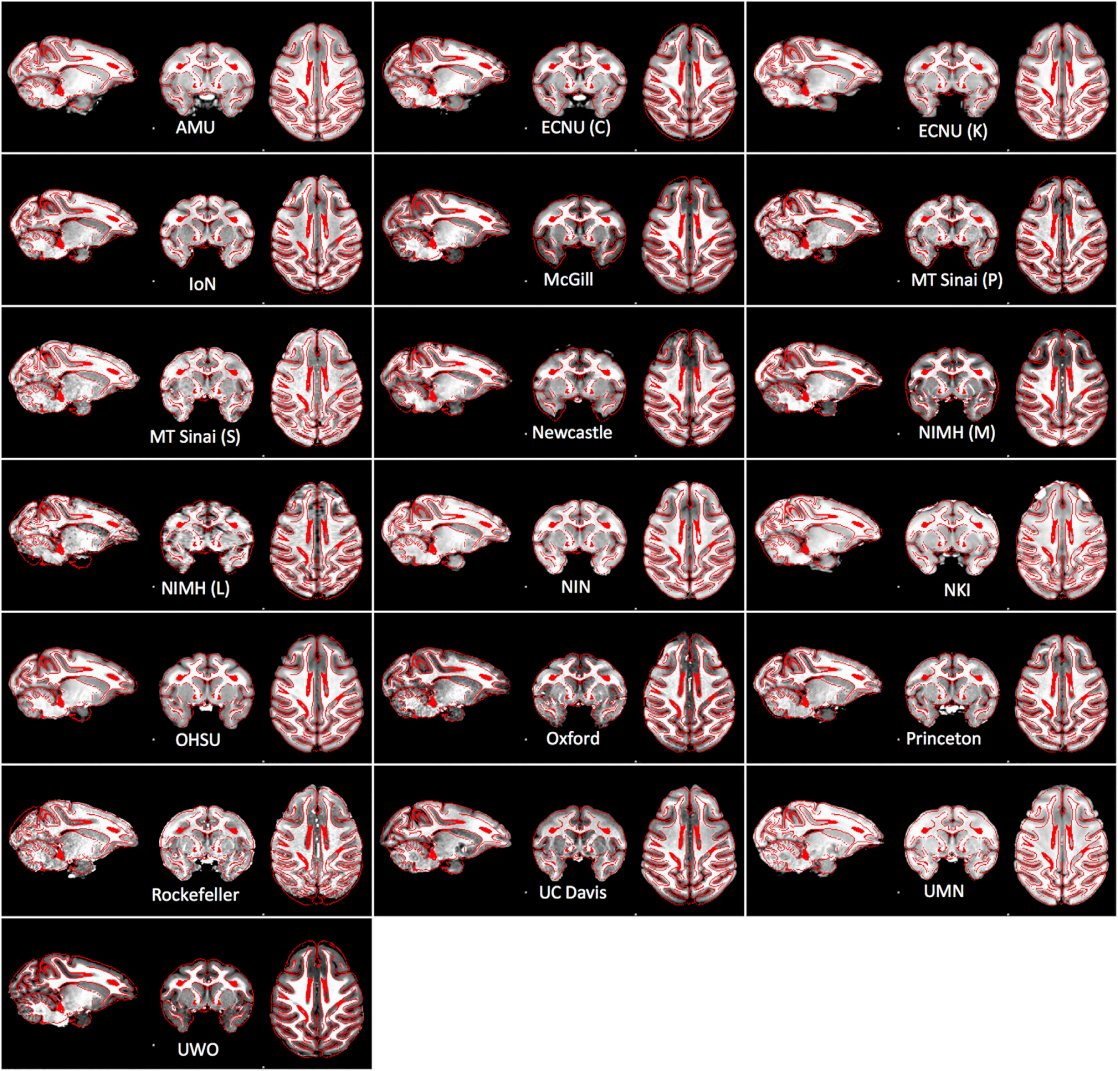
Example Structural Images Example structural images aligned to the common space defined by the NMT template.

**Figure 4:**
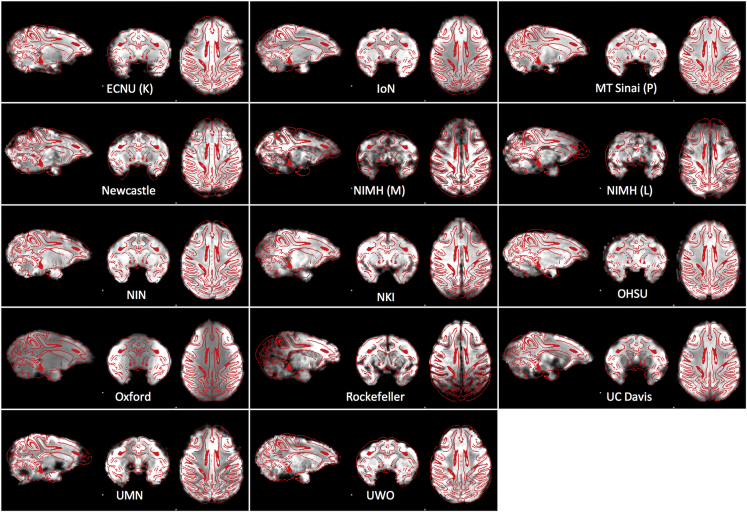
Example Functional Images Example functional images aligned to the common space defined by the NMT template.

## Discussion

### Challenges in the Processing of Non-human Primate Imaging Data

We confront a variety of challenges when trying to adapt well-established methods for human neuroimaging processing to primate data. Beyond the differences between species in tissue contrast, brain shape and size, and type and amount of tissue surrounding the brain, there are significant differences in data collection equipment and acquisition protocols. Non-human primate data are often acquired at very high fields (4.7T, 7T, 9.4T, 11.7T), using some non-standardized arrangement of surface coils. These result in increased variations in image intensity due to B1 inhomogeneity and non-uniform coil coverage and in greater distortion and dephasing due to susceptibility. Another issue is that the equipment and acquisition protocols used are typically customized, resulting in substantial variation in the quality and characteristics of data collected at different sites. Consequently, there is no one-size-fits-all strategy for processing animal data, and researchers need a great deal of flexibility to optimize their pipelines for the data at hand.

Brain extraction and tissue segmentation are more challenging in non-human primate imaging data due to differences in tissue contrast and the nature of structures immediately surrounding the brain. If compromised, these steps in turn can dramatically compromise image registration and normalization procedures as well as temporal de-noising approaches. As of yet, there is no consensus for an optimal solution for each of these processing steps, in part due to the many sources of variation across studies that can differentially impact data characteristics and quality (e.g., anesthesia protocols, coil type, use of contrast agents, magnet strength, animal/rodent type). Additionally, commonly used pre-processing pipelines, used extensively with human neuroimaging datasets, often fail to work properly on non-human primate datasets. As a result, researchers commonly work to optimize individual steps for their datasets outside of traditional workflows, resulting in different pipelines and processing steps across groups. There are efforts underway to form best practices to guide this process and help researchers avoid the need to redefine pipelines themselves (e.g., Seidlitz et al., 2018b, Love et al., 2016); currently, however, it is still necessary for researchers to do so.

### Resources and Solutions

#### Templates and Atlases

A number of macaque templates were created in the last decade, including single-animal templates, e.g., the NeuroMap macaque atlas (M.F. Dubach and D.M. Bowden, 2009, Soc. Neurosci., abstract) and the 3D Digital D99 Template (Reveley et al., 2017), and population-averaged templates based on multiple animals, e.g., 112RM-SL (McLaren et al., 2009), INIA19 (Integrative Neuroscience Initiative on Alcoholism; (Rohlfing et al., 2012), MNI (Montreal Neurological Institute; (Frey et al., 2011), CIVM MRI/DTI atlas (Calabrese et al., 2015), and the most recent NMT (National Institute of Mental Health Macaque Template; (Seidlitz et al., 2018b). In addition, there are surface-based atlases, including the macaque single-subject F99 atlas (Van Essen, 2012, Van Essen, 2002) and the group-average Yerkes19 macaque atlas (Donahue et al., 2016). Data collected in individual macaques can be aligned to these templates using affine and non-linear registration. These templates provide a common anatomical space and coordinate system for specifying specific brain locations and visualizing data collected across days, animals, and laboratories.

Of note, some templates link to volumetric digital brain atlases (Frey et al., 2011, Reveley et al., 2017, Seidlitz et al., 2018b, Saleem and Logothetis, 2012) derived from analysis of histological tissue (Saleem and Logothetis, 2012, Paxinos et al., 1999, Paxinos, 2009). These anatomical parcellations can be warped to individual subjects using standard linear and non-linear registration algorithms (e.g., AFNI’s 3dAllineate and 3dQwarp). Scripts to automate this alignment are available for the single-subject D99 template (https://afni.nimh.nih.gov/pub/dist/atlases/macaque) and the recently published National Institute of Mental Health Macaque Template (NMT; Seidlitz et al., 2018b; https://afni.nimh.nih.gov/NMT). The NMT is a high-resolution (0.25 mm isotropic) T1 template built from *in vivo* scans of 31 young adult macaques. This volume (and accompanying surfaces) is representative of the adult population and provides anatomical detail akin to that of *ex vivo* templates, which require days of scanning to acquire. The NMT is available via the PRIME-DE website as well as on GitHub (https://github.com/jms290/NMT). The database also includes resting-state data from three subjects that have been aligned to the NMT (see NIMH (M) in Table 1). A similar multi-subject template also exists for pre-pubertal rhesus monkeys (Fox et al., 2015); additionally, the publically available UNC-Wisconsin Rhesus Macaque Neurodevelopment Database features a longitudinal dataset that can be used to provide insights into age-related changes in structure (Young et al., 2017).

Other anatomical parcellations have been defined on the surface using the single-subject F99 template (available in Caret; Van Essen et al., 2012), which can be used for analysis on the cortical sheet. For example, the cortical parcellation from Markov et al. (2014) includes quantitative tract-tracing connectivity estimates for a subset of these regions.

#### Improving Skull Extraction, Segmentation, and Registration

A high-quality T1 image with isotropic voxels is important for skull extraction. There are a number of brain extraction algorithms and available tools, e.g., the Brain Extraction Tool (BET in FSL; Smith, 2002), 3dSkullStrip in AFNI (Cox, 1996), the Hybrid Watershed Algorithm (HWA in FreeSurfer; (Ségonne et al., 2004), BSE in BrainSuite (Shattuck and Leahy, 2002), Robust Brain Extraction (ROBEX; Iglesias et al., 2011), Primatologist toolbox (Balbastre et al., 2017), and ANTs (Avants et al., 2011). Most of these tools can be effectively applied to human data; however, the performance is suboptimal and variable in NHP due to the differences in brain structure (e.g., size, adipose tissue, olfactory bulb) and the quality of the T1 image (SNR, inhomogeneous intensity). Accordingly, the parameters and/or related atlas library need to be customized to optimize the brain extraction in NHP. For example, in AFNI, the program “3dSkullStrip” with alternative options “-monkey,” “-marmoset,” and “-surface_coil” is available for brain extraction in NHP. Population brain templates, such as the NMT, can further improve and automate the registration and brain extraction process (Seidlitz et al., 2018b).

Standard segmentation algorithms can separate gray versus white matter, but if the signal is not homogeneous, which is typically the case at higher magnetic fields, segmentation in some parts of the brain will be better than others (especially subcortically). Registration of T2 datasets to T1 structural scans also remains a challenge. Affine or non-linear registration algorithms can work well provided that intermediate scans are available. For instance, a full brain T1 structural scan from the same individual obtained along with T2 images (also with as much coverage of the brain as possible) could be crucial for registering T2 datasets to any of the freely available monkey template brains that are registered to macaque atlases.

One way to reduce or eliminate the manual intervention during brain extraction and tissue segmentation—using only the typically acquired T1 scan—is to rely on priors defined on a high-resolution and high-contrast template. The multi-subject NMT includes manually refined masks of the brain, cortical gray matter, and various tissue types (including blood vasculature; Seidlitz et al., 2018b). Applying the inverse anatomical alignment transformations to the NMT brain mask produces an approximate single-subject mask for brain extraction. A more precise individual brain mask and tissue segmentation can be obtained using the NMT’s representative brain and tissue segmentation masks as priors. The NMT distribution includes scripts that use AFNI and ANTs to perform these mask refinements (as well as morphological analysis). These improvements could be critical for later processing steps for fMRI data. Furthermore, the NMT includes surfaces for visualization of individual subject or group results in a standard coordinate space. Future work could add to these advances, such as tailoring existing surface-based processing pipelines (e.g., CIVET or FreeSurfer) to be specifically used with non-human primate MRI data.

#### Head Motion

Head motion in NHP imaging is an important concern, just as it is in human neuroimaging studies. For the most part, one can apply human imaging motion correction techniques to NHP data directly. However, there are a few concerns with NHP neuroimaging that will be addressed below.

Anesthesia is commonly used in NHP functional neuroimaging, in part due to the lower behavioral and technical demands compared to awake imaging. As reflected by the QAP results, another benefit is that anesthesia dramatically reduces motion artifacts during NHP scanning. However, the use of anesthesia comes with its own set of tradeoffs dealing with how the drugs used interact with neural activity. There are changes in FC patterns due to the particular set and doses of agents used and in comparison to awake imaging (Xu et al., 2018). For this reason, researchers should always assess how anesthesia may, or may not, influence the results of their study before using it. It should be noted that in some studies, anesthesia can be an experimental goal; for example, fMRI imaging in anesthetized macaques can help reveal brain mechanisms of loss of consciousness (Barttfeld et al., 2015).

In awake NHP imaging, the animals are far more likely to create motion artifacts, which need to be addressed during data preprocessing and analyses when they occur. Of note, these artifacts tend to be caused by body movements (Pfeuffer et al., 2007) rather than head movements, as the head is usually fixed and stable. Body movements can cause changes in the magnetic field, making the shimming performed at the beginning of the scan ineffective (Pfeuffer et al., 2007); the monitoring of full body position can be helpful to eliminate motion artifacts (Keliris et al., 2007). Additionally, acclimation to the chair and scanner setup and training to remain still are of great importance in reducing the amount of motion artifacts. As with human neuroimaging best practices, keeping individual scan periods to the shortest necessary for your task will help to reduce motion artifacts. Recent human studies also suggested that movie (NV-fMRI) paradigm may help to reduce head motion relative to resting conditions (e.g., Vanderwal et al., 2015, Alexander et al., 2017). This is also true in awake NHP imaging; for example, in the PRIME-DE NKI site, the mean FD for rest sessions was 0.21 (SD = 0.03), but 0.14 (SD = 0.07) during movie sessions (t = 2.82, p = 0.006, df = 128).

Regarding motion-correction algorithms, those designed for human neuroimaging data perform similarly for NHP data. As such, most groups use SPM, AFNI, ANTs, or FSL software to estimate the motion parameters and remove motion artifacts. The estimates of the movement values can be used as regressors of no interest during the analysis of functional data, if desired. The grayplot, proposed by Power (2017), can be used to illustrate the motion and the de-noising effects. However, as with all neuroimaging data, image distortions or signal drop-out caused movement correction to be suboptimal.

### Next Steps

The PRIME-DE is an ongoing data-sharing consortium stewarded by INDI, which has shared more than 15,000 human imaging datasets over the past decade. As such, we invite new contributions from all investigators in the NHP imaging community, not just those involved in the consortium at the time of the initial release. It is our hope that future contributions will help to capture and promote emerging trends in the NHP community, such as the increasing ability to image during awake states and usage of high-field scanners (e.g., 7.0T), as well as the growing range of species being examined (e.g., marmosets). Additionally, we hope that other data modalities obtained in the NHP community (e.g., electrophysiology) will be shared with higher frequency. Similar to other INDI-based efforts, PRIME-DE is intended to take the first step—establishing a culture for sharing. The logical second step is building toward an optimal infrastructure for sharing. In this regard, it is our hope that open access database and computational platforms will work to increase their support for the needs of NHP imaging. Finally, it is our hope that, building upon the spirit of sharing engendered in PRIME-DE, users will share their resultant statistical maps with one another via venues such as Neurovault (Gorgolewski et al., 2015), which can now handle results from NHP studies.

## STAR★Methods

### Key Resources Table

**Table:** 

REAGENT or RESOURCE	SOURCE	IDENTIFIER
**Software and Algorithms**		

NMT Template	Seidlitz et al., 2018b	https://github.com/TingsterX/PRIME-DE
Preprocessed Connectome Project Quality Assurance Protocol	Shehzad et al., 2015	http://preprocessed-connectomes-project.org/quality-assessment-protocol/
FSL	Smith et al., 2004	https://fsl.fmrib.ox.ac.uk/fsl/fslwiki; RRID: SCR_002823
AFNI	Cox, 1996	https://afni.nimh.nih.gov/; RRID: SCR_005927
FreeSurfer	Fischl, 2012	https://surfer.nmr.mgh.harvard.edu/; RRID: SCR_001847
ANTs	Avants et al., 2011	http://stnava.github.io/ANTs/; RRID: SCR_004757

### Contact for Reagent and Resource Sharing

Further information and requests for resources and reagents should be directed to and will be fulfilled by the Lead Contact, Michael P. Milham (michael.milham@childmind.org).

### Experimental Model and Subject Details

#### Ethics Approval and Consent to Participate

All experimental procedures were approved by local ethics boards prior to any data collection. UK macaque datasets were obtained with Home Office approval and abide with the European Directive on the protection of animals used in research (2010/63/EU). For the NIN Primate Brain Bank/Utrecht University dataset, post-mortem specimens were loaned from the Netherlands Institute of Neuroscience Primate Brain Bank (PBB; http://www.primatebrainbank.org/). No individuals were sacrificed for PBB brain issue. Instead, brains were collected from individuals that died from natural causes or that had to be humanely euthanized for reasons unrelated to the tissue collection.

### Method Details

#### Criteria for Data Contributions

PRIME-DE welcomes contributions from any laboratory willing to openly share multimodal MRI datasets obtained from non-human primates, including but not limited to functional MRI, diffusion MRI and structural MRI. Contributors are responsible for ensuring that any data collected and shared were obtained in accordance with local ethical and regulatory requirements.

There are no set exclusion criteria. We encourage the sharing of all data, independent of quality. This decision is based on the realizations that: 1) there is no consensus on acceptable criteria for movement in functional MRI or diffusion MRI data, 2) high motion datasets are essential to the determination of the impact of motion on reliability, and 3) new approaches continue to be developed to account for movement artifacts. We also encourage submission of data from other modalities (e.g., ASL) or experimental paradigms (e.g., longitudinal data, pharmacologic manipulations) when available.

#### Metadata

Any imaging metadata (e.g., protocol parameters) provided with the data contribution are represented in the BIDS data format. In the case that data are provided in DICOM format, the metadata from the DICOM are used to population the .json file available with BIDS.

Given that this is a retrospective data collection, phenotypic data primarily focuses on basic measures that are relatively standard in the neuroimaging field, as well as those fundamental for analyses and sample characterization. Minimal phenotypic information includes: age, sex, species. The contribution of additional variables that can enhance data usage is encouraged, though not required.

When additional measurements of brain function and behavior are available (e.g., electrophysiology, eye tracking), we will share this data along with the imaging. For any data types that are not yet included in the BIDS format, we will include the relevant metadata in accompanying .csv files; a readme.txt file will facilitate any additional instructions for integration of information. In the long-run, we expect that such specifications will evolve in the BIDS format and we will adopt them accordingly.

Following the model of prior efforts, all contributions are reviewed by the INDI team following upload and corrected as needed to ensure consistent data organization within and across sites. Before open release, each contributing site reviews their reorganized phenotypic records, five random images per imaging modality and their collection-specific narrative for final approval.

#### Alignment to a Common Space

For the purposes of illustration, we depict sample anatomical and functional images (when available) for each contribution to PRIME-DE. Here, we provide a summary of the steps employed for alignment to the common space defined by the NMT template (Seidlitz et al., 2018b), which was essential for creation of Figures 3 and 4 (extracted brains and scripts required for generation of figure are available at: https://github.com/TingsterX/PRIME-DE).

The intensity correction was first applied to T1 images using ANTs ‘N4BiasFieldCorrection’. Then the T1 images were skull stripped using the AFNI 3dSkullstrip with ‘-monkey’ option and ANTs tools by registering the individual head image to NMT head template and then inverse transformed the NMT brain mask into the individual space. The better brain masks were selected and manually corrected if needed. The skull stripped T1 images were then registered to NMT template for the final demonstration.

The functional image was initially skull stripped using the union of the results of ‘bet2′ and ‘3dAutomask’. The T1 brain mask created from the structural processing above was then transformed back to the functional space for further refinement of the functional brain mask for a given subject; this was accomplished using the inverse transform calculated from the transformation from the space of the EPI to that of the high resolution anatomical image (i.e., rigid body transformation). Finally, the functional image was extracted again using the refined brain mask and registered to the T1 image. For the final demonstration, we combined the transformation from functional to anatomical image and the warp from anatomical to template to align functional image to the NMT template.

### Data and Software Availability

#### Data Preparation and Aggregation

PRIME-DE data aggregation is carried out through the International Neuroimaging Data-sharing Initiative (INDI; Mennes et al., 2013); the portal is located at the Neuroimaging Informatics Tools and Resources Clearinghouse (NITRC; http://fcon_1000.projects.nitrc.org/indi/indiPRIME.html).

#### NMT Alignment

Extracted brains and scripts required for generation of Figures 3 and 4 are available at: https://github.com/TingsterX/PRIME-DE.

## Supplemental Information

Table S1. T1 Structural MRI Sequence Information, Related to Table 2 FA, flip angle; TI, inversion time; TE, echo time; ES, echo spacing; BW, bandwidth per pixel; TR, repetition time; PA, parallel acquisition; PF, partial Fourier (half scan); SO, slice orientation; PE, phase encoding direction; RO, read out direction; SL, slice direction. Reconstructed resolution (RR; mm) and image dimensions (RID; px) refer to the images after they have been reconstructed from the k-space data, the matrix size, and resolution used for the acquisition may differ. For these categories, RO, read out direction; PE, phase encoding direction, and SL, slice direction. TA, acquisition time.

Table S2. T2 Structural MRI Sequence Information, Related to Table 2 TE, echo time; TR, repetition time; PE, phase encoding; RO, read out direction; Reconstructed resolution (RR; mm) and image dimensions refer to the images after they have been reconstructed from the k-space data, the matrix size, and resolution used for the acquisition may differ. For these categories, RO, read out direction; PE, phase encoding direction, and SL, slice direction.

Table S3. Functional MRI Sequence Information, Related to Table 2 FA, flip angle; TE, echo time; TR, repetition time; BW, bandwidth per pixel; ES, echo spacing; PA, parallel acquisition; PF, partial Fourier (half scan); PE, phase encoding direction; FS, fat suppression; SO, slice orientation; SA, slice acquisition order; Gap, gap between slices; RO, read out direction; Nacq, number of volumes collected; Ndisc, number of initial volumes discarded by the scanner; TA, acquisition time. Reconstructed resolution (RR; mm) and image matrix (RIM; px) refer to the images after they have been reconstructed from the k-space data, the matrix size, and resolution used for the acquisition may differ. For these categories, RO, read out direction; PE, phase encoding direction, and SL, slice direction.

Table S4. Diffusion MRI Sequence Information, Related to Table 2 FA, flip angle; TE, echo time; TR, repetition time
